# Baseline prolonged venous transit on CT perfusion predicts the post-treatment Brush Sign after successful reperfusion in acute ischemic stroke patients

**DOI:** 10.3389/fneur.2026.1819534

**Published:** 2026-08-28

**Authors:** Derek A. Tsang, Mona Gad, Andrew Cho, Karthik Lalwani, Hamza Salim, Dhairya Lakhani, Cynthia Greene, Manisha Koneru, Adam Dmytriw, Adrien Guenego, Ferdinand Hui, Jeremy Heit, Tobias Faizy, Greg Albers, Hanzhang Lu, Yasmin Aziz, Aakanksha Sriwastaw, Vivek Yedavalli

**Affiliations:** 1 Russell H. Morgan Department of Radiology and Radiological Science, Johns Hopkins Medicine, Baltimore, MD, United States; 2 Department of Neuroradiology, MD Anderson Medical Center, Houston, TX, United States; 3 WVU Rockefeller Neuroscience Institute, Morgantown, WV, United States; 4 Cooper University Health Care, Camden, NJ, United States; 5 Neuroendovascular Program, Massachusetts General Hospital and Brigham and Women’s Hospital, Harvard Medical School, Boston, MA, United States; 6 Universite Libre de Bruxelles, Brussels, Belgium; 7 The Queen's Medical Center, Honolulu, HI, United States; 8 Division of Neuroimaging and Neurointervention, Stanford University, Stanford, CA, United States; 9 Universitatsklinikum Munster, Münster, Germany; 10 University of Cincinnati, Cincinnati, OH, United States

**Keywords:** Brush Sign, intravascular thrombolysis, MRI, prolonged venous transit, stroke

## Abstract

**Background:**

Prolonged venous transit (PVT) on computed tomography perfusion (CTP) indicates impaired macroscopic venous outflow. The Brush Sign on susceptibility-weighted imaging (SWI) reflects microvascular venous congestion and the “no-reflow” phenomenon. We investigated whether pre-treatment PVT independently predicts the post-treatment Brush Sign in patients with anterior circulation acute ischemic stroke with large-vessel occlusion (AIS-LVO) following successful mechanical thrombectomy (MT).

**Methods:**

We retrospectively analyzed patients with anterior-circulation acute ischemic stroke due to large-vessel occlusion who achieved successful reperfusion (modified Thrombolysis in Cerebral Infarction [mTICI] 2b–3) and underwent post-treatment magnetic resonance imaging (MRI). Pre-treatment PVT positivity (Tmax ≥10 s in the superior sagittal sinus and/or torcula) was evaluated on baseline CTP. The primary outcome was the presence of the Brush Sign on follow-up SWI. A multivariable logistic regression model evaluated the independent association between PVT and the Brush Sign, adjusting for age, admission National Institutes of Health Stroke Scale (NIHSS), intravenous thrombolysis, final infarct volume, mTICI score, and baseline Alberta Stroke Program Early CT Score (ASPECTS).

**Results:**

Of 187 included patients, 29 (15.5%) demonstrated the Brush Sign. Pre-treatment PVT + was significantly more prevalent in patients who developed the Brush Sign (48.3% vs. 27.2%, *p* = 0.02). In the adjusted multivariable model (N = 130), PVT + remained a strong, independent predictor of the Brush Sign (OR 3.82, 95% CI 1.32–11.77, p = 0.02). The association remained robust across sensitivity analyses accounting for MRI timing, complete reperfusion, occlusion segment, and suspected stroke etiology.

**Conclusion:**

Pre-treatment PVT independently predicts the post-treatment Brush Sign in AIS-LVO patients despite successful macrovascular reperfusion. This finding links macroscopic venous congestion to downstream microvascular failure, highlighting PVT as an easily accessible imaging biomarker for anticipating the no-reflow phenotype.

## Introduction

Acute ischemic stroke due to large-vessel occlusion (AIS-LVO) in the anterior circulation remains a significant cause of morbidity and mortality (1). While advances in reperfusion therapies, specifically mechanical thrombectomy (MT), have revolutionized stroke care, a significant subset of patients continues to experience poor functional recovery despite achieving successful macrovascular recanalization (mTICI 2b-3) (2–4). This discrepancy is increasingly attributed to the “no-reflow” phenomenon, in which microvascular perfusion fails to normalize despite the restoration of flow in the primary occluded artery (5).

Cerebral venous outflow (VO) has emerged as an important prognostic imaging biomarker for evaluating the completeness of cerebral tissue reperfusion. Recent studies have highlighted the importance of VO profiles as an integral component of the cerebral collateral cascade for individuals presenting with AIS-LVO (6–11). Impaired VO has been associated with post-procedural cerebral edema, hemorrhagic transformation, and poor clinical outcomes (8, 12–15). In contrast, patients treated with MT are more likely to experience favorable clinical outcomes when their VO is favorable (13).

Computed tomography perfusion (CTP) is commonly utilized in several medical facilities for triaging patients with AIS-LVO for MT (16–18). Prolonged venous transit (PVT), defined as the presence of time to maximum (Tmax) ≥ 10s in the superior sagittal sinus (SSS), torcula, or both, has been validated as a robust surrogate for impaired VO and is easily interpreted visually using post-processed Tmax maps on CTP. Previous studies have demonstrated that pre-treatment PVT positivity strongly correlates with 90-day mortality in successfully reperfused AIS-LVO patients (9, 12, 19–23).

On imaging, the “Brush Sign,” characterized by prominent asymmetric, enlarged medullary veins on susceptibility-weighted imaging (SWI), is a recognized marker of increased oxygen extraction fraction and deep venous congestion (24). Recent literature has demonstrated that the presence of the Brush Sign is associated with worse clinical and radiographic outcomes, including larger infarct growth and poorer functional recovery (24–28). Thus, the Brush Sign is believed to represent a downstream microvascular manifestation of the no-reflow phenotype. However, it remains unknown whether proximal venous congestion, as measured by pre-treatment PVT on CTP, can predict persistent microvascular venous congestion (the Brush Sign) following successful MT.

In this study, we investigated the relationship between pre-treatment PVT and the presence of the Brush Sign on post-treatment MRI. We hypothesized that PVT positivity on baseline CTP would serve as an independent imaging biomarker for the presence of the Brush Sign, persisting even after successful macrovascular reperfusion.

## Methods

### Study design and population

From a prospective registry of patients with acute ischemic stroke initiated on 1 September 2017, we retrospectively identified eligible patients through 29 May 2023, the date of the last eligible patient included in the finalized study dataset. The inclusion criteria were as follows: (1) confirmed anterior circulation large-vessel occlusion (ICA, M1, or M2 segments) on baseline computed tomography angiography (CTA); (2) successful reperfusion following mechanical thrombectomy (MT), defined as a modified Thrombolysis in Cerebral Infarction (mTICI) score of 2b, 2c, or 3; and (3) available post-treatment magnetic resonance imaging (MRI), specifically including susceptibility-weighted imaging (SWI) and diffusion-weighted imaging (DWI). Patients who did not achieve successful macrovascular reperfusion or did not undergo post-treatment MRI were excluded from the analysis.

### Clinical and procedural data collection

Baseline clinical data were collected by certified neurologists or nurse practitioners at the time of the clinical encounter and were retrospectively extracted from the electronic medical record. These data included patient demographics, stroke risk factors (hypertension, diabetes, and atrial fibrillation), admission National Institutes of Health Stroke Scale (NIHSS) score, and administration of intravenous thrombolysis (IVT). The final post-treatment mTICI score was determined by the treating neurointerventionalist and subsequently verified retrospectively by consensus between two expert. Suspected stroke etiology was classified according to the Trial of Org 10,172 in Acute Stroke Treatment (TOAST) criteria as large-artery atherosclerosis, cardioembolism, small-vessel occlusion, other determined etiology, or undetermined etiology.

### Imaging data collection and PVT assessment

Imaging data obtained from contemporaneous radiology reports were retrospectively reexamined and validated by an experienced neuroradiologist, who also obtained post-processed imaging data and performed additional retrospective imaging assessments.

Alberta Stroke Program Early CT scores (ASPECTS) were calculated from pre-treatment non-contrast CT by an experienced neuroradiologist (V.Y.; 11 years of experience). The presence, segment, and laterality of large-vessel occlusion were identified based on baseline CTA by an experienced neuroradiologist (V.Y.). The presence of complications, such as hemorrhagic transformation, as defined by the European Cooperative Acute Stroke Study (ECASS 2) trial, was assessed on follow-up non-contrast CT or susceptibility-weighted MRI within 48 h of initial presentation.

Whole-brain pre-treatment CTP was performed on the Siemens Somatom Force (Erlangen, Germany) with the following parameters: 70 kVP, 200 effective mAs, rotation time 0.25 s, average acquisition time 60 s, collimation 48 × 1.2 mm, pitch of 0.7, and four-dimensional range 114 mm × 1.5 s. Pre-treatment CTP images were then postprocessed using RAPID commercial software (IschemaView, Menlo Park, CA, USA) to generate quantitative relative cerebral blood flow (rCBF) volumes, Tmax volumes, and qualitative Tmax maps.

**Figure 1:**
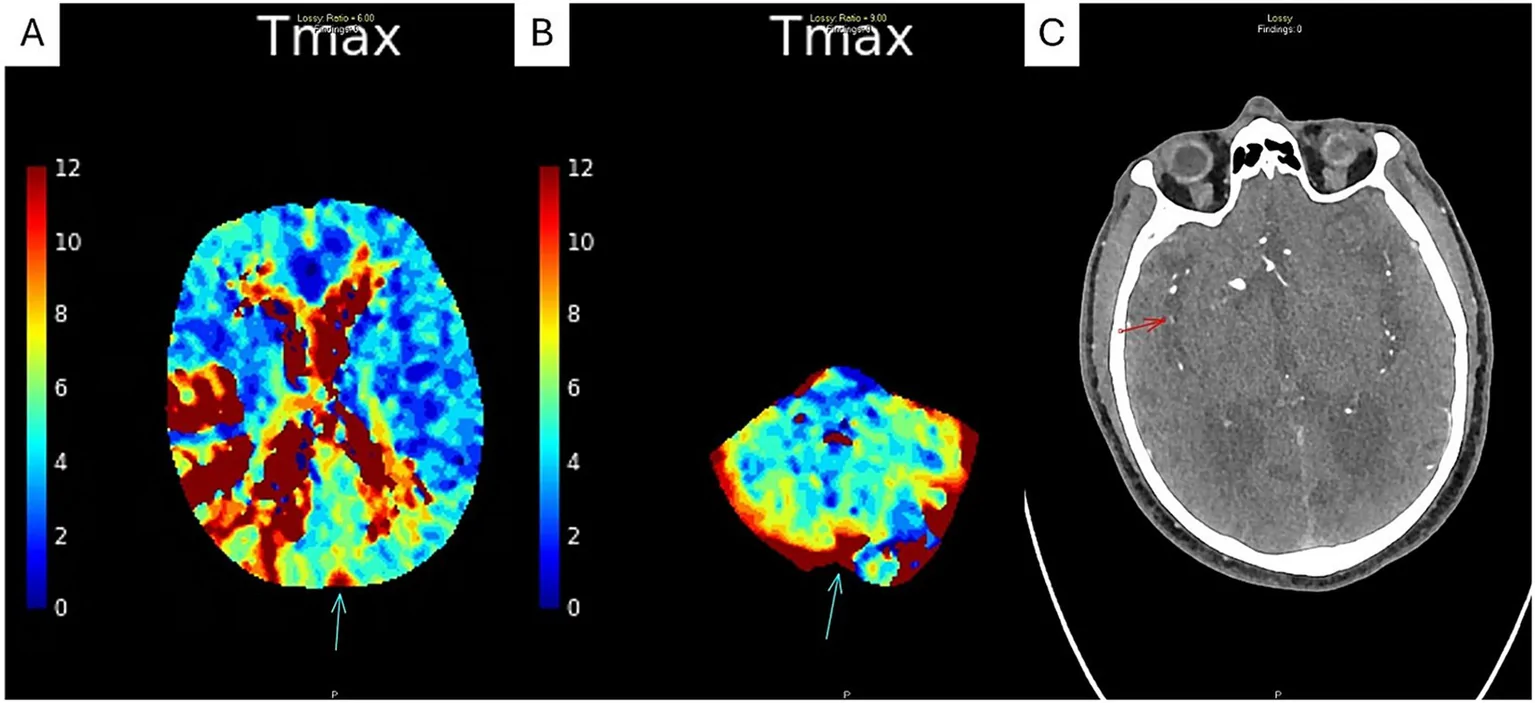
Pre-treatment CTP demonstrating PVT+. Imaging from an elderly male presenting with acute right M2 occlusion. His admission National Institutes of Health Stroke Scale score was 19. The 90-day modified Rankin Scale score was 6. Tmax perfusion maps showing prolonged venous transit in **(A)** the superior sagittal sinus (blue arrow) and **(B)** the torcula (blue arrow). Computed tomography angiography **(C)** highlighting the right M2 occlusion (red arrow).

Based on qualitative Tmax maps, PVT was assessed within the posterior SSS at the level of the lateral ventricle occipital horns to incorporate more proximal venous drainage, as well as at the torcula to account for deep venous drainage patterns. PVT positivity was defined as a Tmax ≥10 s within the posterior SSS, torcula, or both areas based on the timing key adjacent to the image. PVT assessments were performed by board-certified neuroradiologists (V.Y., 11 years of working experience, and D.A.L., 6 years of working experience), blinded to the clinical information. Discrepancies were resolved by consensus review. The final infarct volume was quantified using post-treatment follow-up DWI. A representative example of a patient demonstrating pre-treatment PVT positivity on CTP Tmax maps is shown in Figure 1.

### Outcome measure: Brush Sign assessment

**Figure 2:**
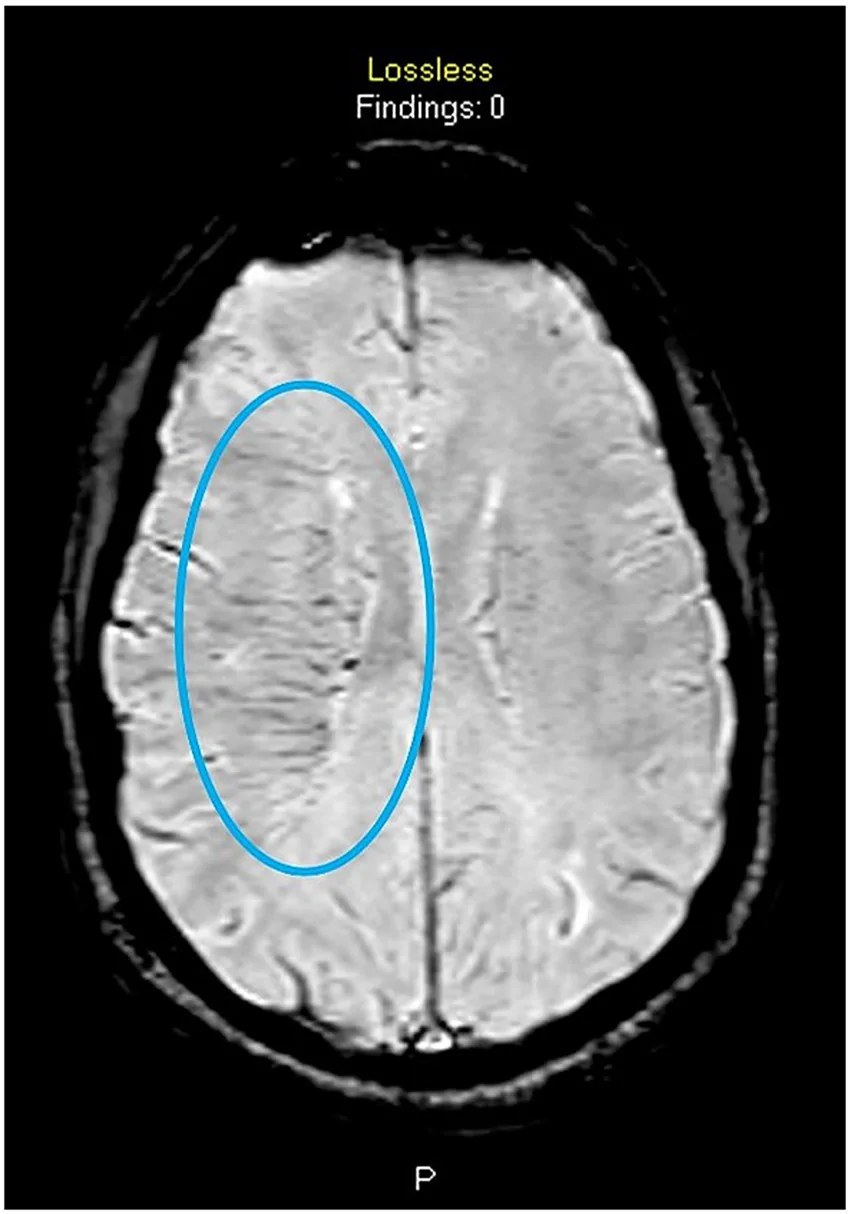
Post-treatment SWI demonstrating the Brush Sign. Imaging from a 43-year-old female who presented with a proximal ICA occlusion. Her admission National Institutes of Health Stroke Scale score was 17. The 90-day modified Rankin Scale score was 4. Following successful macroscopic recanalization, follow-up SWI revealed the prominent right-sided Brush Sign (blue oval).

The primary outcome of this study was the presence of the Brush Sign on post-treatment MRI. The Brush Sign was assessed on post-treatment SWI and was defined as the visible presence of enlarged medullary veins in the parenchyma. For this analysis, the Brush Sign was treated as a binary outcome (positive or negative). Brush Sign assessments were performed by board-certified neuroradiologists (V.Y., 11 years of working experience, and D.A.L., 6 years of working experience), blinded to the clinical information. Discrepancies were resolved by consensus review. A representative example of the Brush Sign, demonstrating prominent, enlarged medullary veins on post-treatment SWI, is shown in Figure 2. The interval from baseline stroke imaging to follow-up MRI was calculated in calendar days based on the dates of the initial imaging examination and the MRI containing the SWI sequence.

## Statistical analysis

The primary objective of this study was to assess the independent association between pre-treatment PVT positivity and the post-treatment presence of the Brush Sign. Descriptive statistics were used to summarize baseline clinical, procedural, and imaging characteristics stratified by Brush Sign status. Continuous variables are reported as medians with interquartile ranges and were compared using the Wilcoxon rank-sum test. Categorical variables are reported as frequencies and percentages and were compared using Pearson’s chi-square test or Fisher’s exact test when expected cell counts were <5.

To determine whether PVT positivity was independently associated with the Brush Sign, a multivariable logistic regression model was constructed. Covariates were selected *a priori* on the basis of clinical relevance and included age, admission NIHSS, intravenous thrombolysis, mTICI reperfusion grade, follow-up DWI infarct volume, and baseline ASPECTS. The model was fitted using complete cases for all included covariates. Patients included in and excluded from the complete-case model were compared using the same univariable methods described above to assess potential selection bias. The results are reported as odds ratios with 95% confidence intervals. No automated or significance-based variable-selection procedure was used.

Interrater agreement for PVT and Brush Sign assessment was evaluated using Cohen’s *κ*.

The interval from baseline stroke imaging to follow-up MRI was calculated in terms of calendar days because follow-up MRI timing was recorded by date. MRI timing was compared according to Brush Sign status using the Wilcoxon rank-sum test. In a sensitivity analysis, a parsimonious logistic regression model evaluated the association between PVT and the Brush Sign after adjusting for age, admission NIHSS, complete reperfusion, defined as mTICI 3 vs. mTICI 2b/2c, and the interval from baseline imaging to follow-up MRI. Additional sensitivity analyses evaluated occlusion segment and suspected stroke etiology, classified according to TOAST criteria, as categorical covariates. Their overall contributions were assessed using likelihood-ratio tests. A separate sensitivity analysis dichotomized reperfusion status as complete reperfusion (mTICI 3) vs. mTICI 2b/2c.

Exploratory secondary analyses evaluated discharge NIHSS, discharge mRS, and 90-day mRS according to PVT and Brush Sign status. Favorable functional outcome was defined as mRS 0–2. Separate multivariable logistic regression models evaluated the associations between PVT and the Brush Sign and a favorable 90-day outcome after adjusting for age and admission NIHSS.

Statistical significance was set at a two-sided *p*-value of <0.05. All statistical analyses were conducted using R software, version 4.4.2.

## Results

### Baseline demographics and clinical characteristics

A total of 187 patients met the inclusion criteria for anterior circulation LVO with successful mechanical thrombectomy (mTICI 2b-3) and available post-treatment MRI. Of these, 29 patients (15.5%) demonstrated the Brush Sign on post-treatment SWI, while 158 patients (84.5%) did not. Baseline demographics and clinical characteristics were well-balanced between the two cohorts (Table 1). There were no statistically significant differences between the Brush Sign negative and positive groups in median age (70 vs. 66 years, *p* = 0.18), proportion of male patients (56.3% vs. 48.3%, *p* = 0.42), or medical comorbidities, including hypertension, atrial fibrillation, and diabetes. Stroke severity upon presentation was also similar, with no significant difference in median admission NIHSS (15 vs. 13, *p* = 0.17) or rates of IV tPA administration (36.1% vs. 31.0%, *p* = 0.60).

**Table 1:** Baseline demographics, clinical characteristics, and radiographic outcomes.

Variables	Brush Sign (−) (*N* = 158)	Brush Sign (+) (*N* = 29)	*P*-value
Demographics and medical history			
Age, median (IQR)	70 (62–78)	66 (60–72)	0.18
Male sex, n (%)	89 (56.3%)	14 (48.3%)	0.42
Hypertension, n (%)	130 (82.3%)	20 (69.0%)	0.10
Atrial fibrillation, n (%)	64 (40.5%)	13 (44.8%)	0.66
Diabetes, n (%)	46 (29.1%)	5 (17.2%)	0.19
Clinical presentation and treatment			
Admission NIHSS, median (IQR)	15 (11–20)	13 (8–19)	0.17
IV tPA administered, n (%)	57 (36.1%)	9 (31.0%)	0.60
Radiographic and procedural outcomes			
Pre-treatment PVT positive, n (%)	43 (27.2%)	14 (48.3%)	0.02*
Baseline ASPECTS, median (IQR)	9.0 (8.0–10.0)	9.0 (8.0–10.0)	0.78
Reperfusion grade (mTICI), n (%)			0.07†
2b	45 (28.5%)	11 (37.9%)	
2c	15 (9.5%)	6 (20.7%)	
3	98 (62.0%)	12 (41.4%)	
Final infarct volume (DWI), median (IQR), mL	22 (8–62)	35 (10–131)	0.16
Hemorrhagic transformation, n (%)	68 (43.0%)	15 (51.7%)	0.47

*Indicates statistical significance (*p* < 0.05). †Indicates a trend toward significance (*p* < 0.10).

### Radiographic and procedural outcomes

Interrater agreement was moderately good for both PVT and Brush Sign assessment (Cohen’s *κ* = 0.64 and 0.60, respectively). Radiographic and procedural variables stratified by Brush Sign status are also summarized in Table 1. Pre-treatment PVT + was significantly more prevalent in patients who subsequently developed the Brush Sign compared with those who did not (48.3% vs. 27.2%, *p* = 0.02). Median baseline ASPECTS was identical between the two groups (median 9.0, *p* = 0.78). Patients who developed the Brush Sign demonstrated a trend toward lower rates of complete (mTICI 3) reperfusion (41.4% vs. 62.0%, *p* = 0.07). In a sensitivity model dichotomizing reperfusion status as mTICI 3 vs. mTICI 2b/2c, complete reperfusion was associated with lower odds of the Brush Sign (OR, 0.42; 95% CI, 0.18–0.98; *p* = 0.045). PVT remained independently associated with the Brush Sign in this model (OR, 3.45; 95% CI, 1.40–8.49; *p* = 0.007). While median final infarct volume on follow-up DWI was larger in the Brush Sign positive cohort (35 mL vs. 22 mL), this difference was not statistically significant (*p* = 0.16). Furthermore, rates of hemorrhagic transformation were similar between the two groups (51.7% vs. 44.3%, *p* = 0.47).

The interval from baseline imaging to follow-up MRI was available for 185 patients, with a median of 2 days (IQR, 1–4). MRI timing did not differ between patients without and with the Brush Sign (median, 2 days [IQR, 1–4] vs. 2 days [IQR, 1–3.25], respectively; *p* = 0.56). In a sensitivity model additionally adjusting for MRI timing, the interval to MRI was not associated with the Brush Sign (OR, 0.94 per day; 95% CI, 0.78–1.13; *p* = 0.50), while PVT remained independently associated with the Brush Sign (OR, 3.61; 95% CI, 1.45–8.97; *p* = 0.006).

Neither occlusion segment nor suspected stroke etiology was significantly associated with the Brush Sign in adjusted sensitivity analyses (global *p* = 0.59 and *p* = 0.70, respectively). PVT remained independently associated with the Brush Sign after adjusting for occlusion segment (OR, 3.35; 95% CI, 1.32–8.55; *p* = 0.011) and suspected stroke etiology (OR, 3.37; 95% CI, 1.36–8.35; *p* = 0.009).

### Multivariable predictors of the Brush Sign

A multivariable logistic regression analysis was conducted in 130 patients with complete data for all included covariates (Table 2). Of the 57 patients excluded from the complete-case model, 56 lacked quantitative follow-up DWI infarct-volume measurements, and one additional patient lacked an admission NIHSS score. Patients included in the complete-case model did not differ significantly from those excluded with respect to age, admission NIHSS, PVT positivity, Brush Sign prevalence, IV thrombolysis, mTICI reperfusion grade, discharge mRS, or 90-day mRS. After adjusting for age, admission NIHSS, IV tPA administration, mTICI reperfusion grade, final infarct volume, and baseline ASPECTS, pre-treatment PVT + remained a strong and statistically significant independent predictor of the Brush Sign (OR 3.82, 95% CI 1.32–11.77, *p* = 0.02). Of note, neither the specific grade of successful reperfusion (mTICI 2c or 3 vs. 2b) nor the final infarct volume was a significant predictor of the Brush Sign in the adjusted model.

**Table 2:** Multivariable logistic regression for prediction of Brush Sign (*N* = 130).

Variables	Odds ratio (OR)	95% confidence interval	*P*-value
PVT+	3.82	1.32–11.77	0.02*
Age	0.97	0.93–1.01	0.15
Admission NIHSS	0.95	0.88–1.03	0.21
IV tPA administered	0.52	0.15–1.60	0.28
mTICI score (Ref: 2b)			
mTICI 2c	0.77	0.14–3.46	0.74
mTICI 3	0.40	0.13–1.24	0.11
Follow-up DWI volume	1	1.00–1.01	0.47
ASPECTS score	1.04	0.78–1.42	0.80

*Indicates statistical significance (*p* < 0.05).

### Exploratory functional outcomes

Discharge NIHSS, discharge mRS, and 90-day mRS were available in 182, 185, and 146 patients, respectively. PVT + patients had higher discharge NIHSS, discharge mRS, and 90-day mRS than PVT − patients (all *p* ≤ 0.001). Brush Sign+ patients had higher discharge NIHSS than Brush Sign− patients (median, 11 [IQR, 5–16] vs. 4 [IQR, 1–9]; *p* = 0.002), although discharge and 90-day mRS did not differ significantly in unadjusted analyses. After adjusting for age and admission NIHSS, PVT + was associated with lower odds of a favorable 90-day outcome (OR, 0.43; 95% CI, 0.19–0.94; *p* = 0.034), as was the Brush Sign (OR, 0.31; 95% CI, 0.11–0.85; *p* = 0.024).

## Discussion

In this study, we found that pre-treatment PVT was significantly and independently associated with the presence of the Brush Sign on post-treatment MRI in patients with anterior circulation AIS-LVO who achieved successful reperfusion. More specifically, PVT + patients had nearly four times the odds of exhibiting the Brush Sign compared to PVT − patients (adjusted OR 3.82, 95% CI 1.32–11.77, p = 0.02). Notably, this association remained significant after adjusting for baseline stroke severity, final infarct volume, and mTICI reperfusion grade.

Our findings provide important neuroimaging evidence linking macro-vascular venous congestion with microvascular failure. PVT positivity serves as a surrogate marker of increased resistance within the proximal venous drainage system, likely exacerbated by elevated interstitial pressure and early ischemic tissue edema (12, 19, 29). The Brush Sign, visible on SWI, reflects an increased concentration of deoxyhemoglobin in the deep medullary veins due to a high oxygen extraction fraction in the setting of persistent hypoperfusion (24).

Multiple recent studies have established that the post-treatment Brush Sign is an indicator of poor prognosis, correlating with worse functional outcomes and increased tissue infarction (24–28). Exploratory analyses also demonstrated associations between both PVT and the Brush Sign and reduced odds of a favorable 90-day outcome after adjusting for age and admission NIHSS. However, these secondary analyses were limited by incomplete follow-up and were not the primary focus of the study. By demonstrating that PVT + is strongly associated with the presence of this detrimental Brush Sign, our findings position PVT as a critical, early upstream indicator of impending microvascular failure. This strong independent association points to a progressive cascade of venous congestion. Patients who present with pre-treatment PVT may already have an overwhelmed venous outflow tract, which could contribute to downstream micro-perfusion. Even after the arterial clot is successfully removed, this persistent venous congestion may manifest radiographically as the Brush Sign and contribute to the “no-reflow” phenomenon.

The persistence of the association between PVT and the Brush Sign after adjusting for reperfusion grade and final DWI infarct volume is particularly noteworthy. While previous investigations have shown that decreased quantitative cerebral blood volume and high hypoperfusion intensity ratios correlate with poor clinical outcomes, our data suggest that PVT captures a distinct pathophysiological mechanism (16, 30–33). The Brush Sign is not simply a byproduct of a larger stroke core or suboptimal (mTICI 2b) recanalization; rather, it is a specific manifestation of impaired venous hemodynamics (19, 20, 23). This aligns with recent literature demonstrating that unfavorable VO profiles predict futile recanalization and higher odds of mortality independent of arterial collateral status (12). The lower frequency of complete reperfusion among patients with the Brush Sign also merits consideration. In a sensitivity analysis, mTICI 3 was associated with lower odds of the Brush Sign compared with mTICI 2b/2c, suggesting that more complete macrovascular reperfusion may reduce, but does not eliminate, the risk of persistent post-treatment venous abnormalities. Importantly, PVT remained independently associated with the Brush Sign after accounting for complete reperfusion.

These findings carry significant clinical implications. The assessment of PVT on CTP-derived Tmax maps is rapid, binary, and requires minimal specialized neuroradiological training (19). By identifying PVT + patients upon presentation, clinicians may be able to proactively identify individuals at high risk for microvascular no-reflow (Brush Sign positivity). This information may support intensified post-thrombectomy monitoring and help identify patients for future studies of individualized hemodynamic, anti-edema, or microcirculatory interventions (34–36). However, the present observational findings do not establish a specific management strategy.

Our study is not without limitations. The retrospective design introduces inherent selection biases, particularly because our inclusion criteria necessitated a post-treatment MRI. Thus, patients who were too clinically unstable to undergo an MRI or who died early in their hospital course were excluded, which may underestimate the true prevalence of both PVT and the Brush Sign in severe stroke cohorts. In addition, the primary multivariable model was restricted to complete cases, predominantly because quantitative follow-up DWI infarct volume was unavailable in 56 patients, which may have introduced selection bias and reduced statistical power. Furthermore, the Brush Sign was treated as a binary variable; future studies could employ a graded scoring system to assess the severity of venous prominence. Although MRI timing did not differ by Brush Sign status and did not materially affect the association between PVT and the Brush Sign, follow-up MRI was obtained according to clinical practice rather than at a standardized post-treatment interval. Short-term outcomes were assessed at hospital discharge rather than at a standardized 14-day time point, and serial post-treatment CT ASPECTS was not systematically obtained. Recurrent ischemic events were not systematically captured in the registry and therefore could not be reliably evaluated.

In conclusion, pre-treatment PVT on CTP is strongly and independently associated with the post-treatment Brush Sign in AIS-LVO patients who achieve successful mechanical thrombectomy. These results reinforce the critical role of venous hemodynamics in the cerebral collateral cascade and suggest that PVT is a valuable, easily accessible imaging biomarker for identifying patients at risk of the microvascular no-reflow phenotype. Future prospective, multicenter studies are warranted to validate these findings and to evaluate whether VO-guided interventions can improve functional outcomes in this high-risk population.

## Data Availability

The raw data supporting the conclusions of this article will be made available by the authors, without undue reservation.
